# Inertial navigation algorithm for trajectory of front-wheel walker estimation

**DOI:** 10.1016/j.heliyon.2019.e01896

**Published:** 2019-06-15

**Authors:** Quang Vinh Doan, Duy Duong Pham

**Affiliations:** a The University of Danang - University of Science and Technology, Danang, Vietnam; b The University of Danang - University of Technology and Education, Danang, Vietnam

**Keywords:** Mechanical engineering, Electrical engineering, Inertial sensor, Walker's trajectory, Front-wheel walker, Inertial navigation, IMU

## Abstract

In this paper, we propose a system for trajectory of walker estimation. The system consists of an inertial measurement unit (IMU) and two encoders attached to a front-wheel walker. The IMU is employed to estimate the trajectory of the walker while the encoders are used to update the trajectory of the walker during rolling on the floor. Three update equations are proposed: quaternion update using the vertical vector, quaternion update using the yaw angle of the walker and position update using encoders. We implemented an experiment which focused on four walking styles of: continuous rolling, step by step rolling, complete lifting and 2 back tips lifting. Results of the experiment show the appropriateness of proposed update equations in all cases in general and in continuous rolling in particular.

## 1 Introduction

Population ages 65 and above [1] brings on more health problems like decreasing mobility/stability, entailing a heavy demand on the health care system [2, 3]. Among people in this age group, using a walker to support walking and physical rehabilitation [4, 5, 6] is common. Additionally, walkers shown in a study in [7] not only interfere with rehabilitation outcome but also, in some cases, reduce the rehabilitation period. There are three kinds of walker: standard walker, front-wheeled walker, and four-wheeled walker (rollator walker) [8].

In order to speed up rehabilitation of people aided with a walker, their needs should be attentively assessed [9]. The tests identify such demands including of walking speed which is proved to be associated with survival rates of adults [10], an important measure in comprehensive geriatric assessment [11], and a responsive measure for patients undergoing short-term rehabilitation [12].

Walking parameter can be estimated via standard tests, with and without walking aids, such as 6-minutes walking test [13], 50-foot walking test, 30-second chair standing test [14] and the timed up and go [15]. In these tests, only average walking speed is computed but not frequently re-evaluated since the tests are usually performed inside hospitals.

Recently, low cost IMU (accelerometer, gyroscope and magnetometer) has been successfully utilized in motion tracking. Due to their acceptable accuracy, low cost, light weight, compact in size and easy to use, these wearable IMU rapidly become one of the most promising solutions for motion tracking. The motion tracking can be used for gait analysis [16, 17, 18], pedestrian navigation [19], control system of industrial robot trajectory [20, 21, 22] and sport [23, 24].

We proposed a comprehensive and portable measurement system to circumvent such drawbacks. Our system consists of a front-wheel walker with an IMU used to estimate trajectory of lifting movement, and two encoders used to estimate trajectory of rolling movement [16]. Walking parameters of users are derived from a trajectory of walker, which is formed by combining estimates from both the IMU and two encoders.

We then employ a Kalman filter-based inertial navigation algorithm to estimate trajectory of both lifting and rolling movements. Trajectory estimation is then updated by data from encoders. Besides, we propose a method to improve the correlation between the IMU and the walker. It is said that, the method is more effective than that mentioned in [16] which assumed a coincidence between the moving direction of walker and the $x_{b}$ axis of body coordinate system. However, the assumption is not completely true. As can be seen clearly, the accuracy of the system depends on accuracy of the relationship estimation. The advantage of our method lies in the design of experiment which allows us to estimate the relationship easily and more accurately than those in [16].

This paper is organized as below. Section 2 shows the overview of the proposed system. The algorithm for the rotation matrix between the IMU and the walker is illustrated in Section 3. In Section 4, the inertial navigation algorithm using Kalman filter is presented. The measurement update equations using encoders is built in Section 5. Finally, the experiments and results to verify the accuracy of the proposed method are shown in Section 3.

## 2 Methods

### 2.1 Overview System

**Fig. 1:**
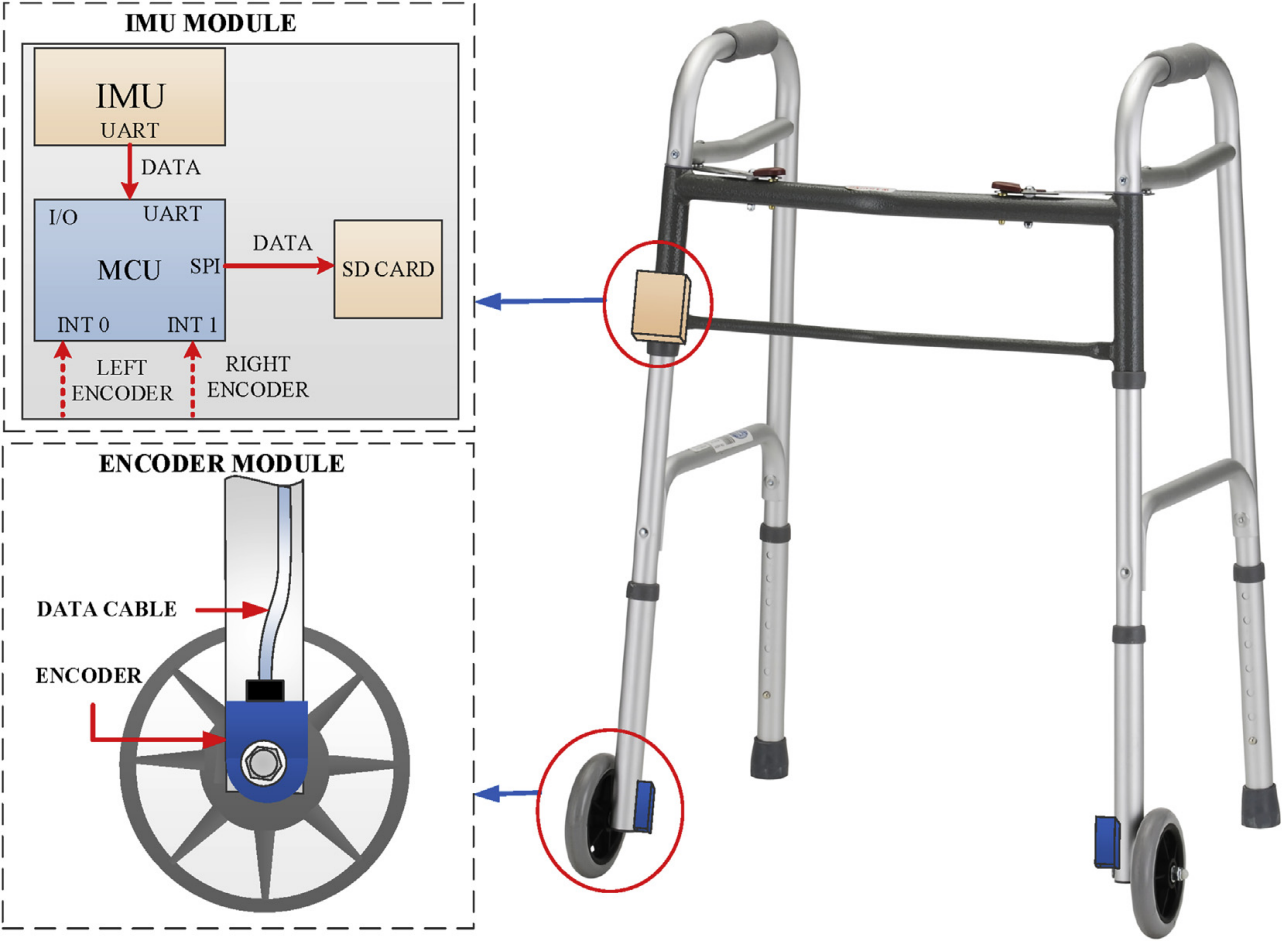
Proposed walker system with an IMU unit and encoder modules.

Our proposed system of a front-wheel walker is shown in Fig. 1. For a standard walker, encoders are not employed since there is no wheel. An IMU module consisting of an IMU (Xsens Mti 1), micro SD card and a microcontroller is attached to the frame of walker. The IMU contains a three-axis accelerometer and a three-axis gyroscope with 100 Hz sampling frequency. The encoders with 1024 pulses per revolution are attached to the wheels.

**Fig. 2:**
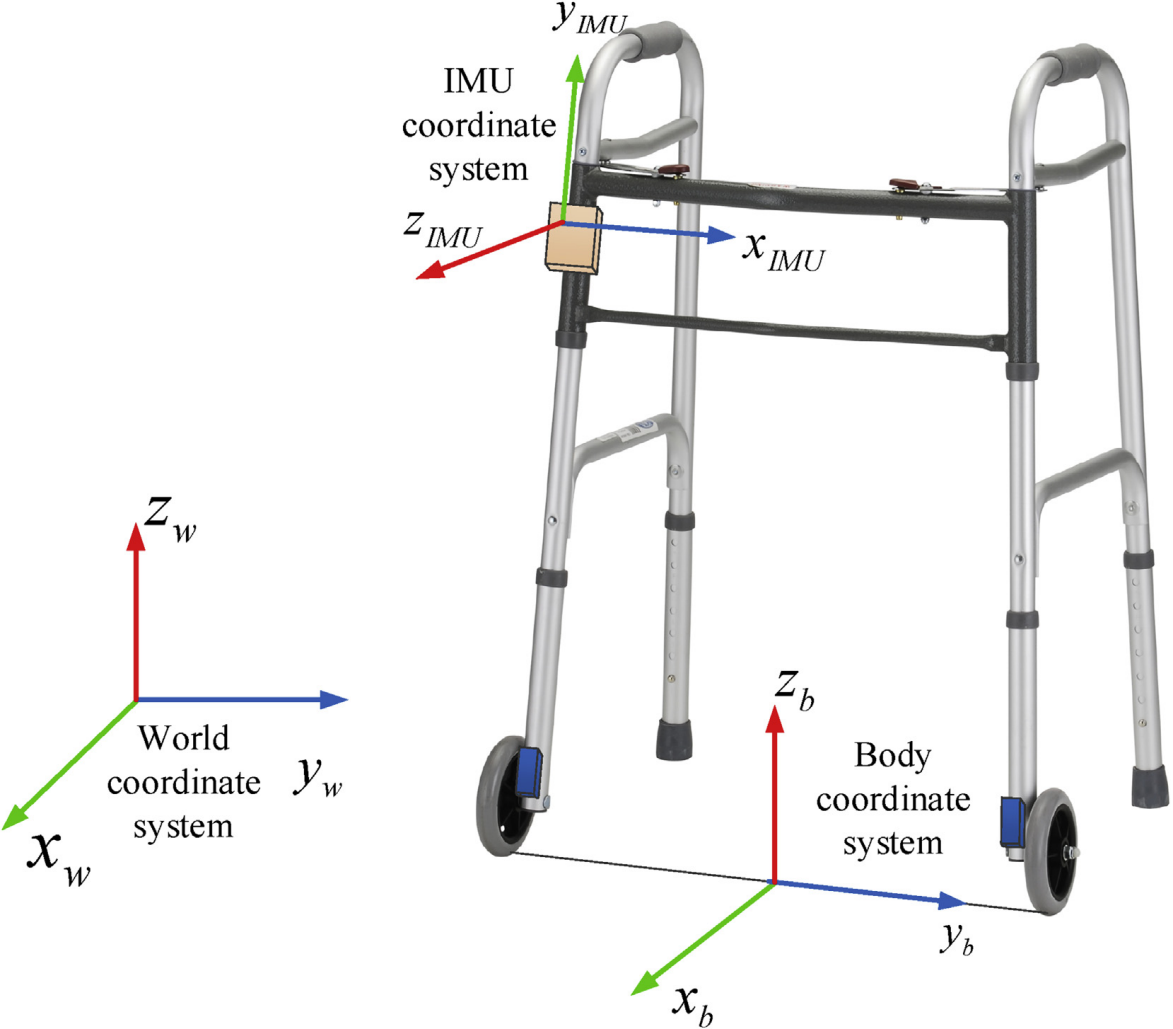
IMU, body and world coordinate systems.

An IMU coordinate system (ICS), a body coordinate system (BCS) and a world coordinate system (WCS) are mentioned in this paper (see Fig. 2). The x axis of BCS coincides with the front direction of the walker while the y axis is on the line connecting the bottom of two wheels and the origin is set at the midpoint of line as in Fig. 2. Thus, the z axis of BCS is pointing upward when the wheel are rolling on the horizontal floor. The z axis of the WCS is pointing upward while the x and y axes are chosen arbitrarily. The origin of the WCS is assumed to be on the floor. The notation ${\left[a\right]}_{w}$ (${\left[a\right]}_{b}$ or ${\left[a\right]}_{I}$) is used to denote that vector a is represented in the world (body or IMU) coordinate system.

The relationships consisting of a translation vector $T_{I}^{b}$ and a rotation matrix $C_{I}^{b}$ form ICS to BCS are necessary for walker's position computing from the IMU's position. In this circumstance, the translation vector can be measured by a ruler and the rotation matrix is estimated by an algorithm as in Section 3.

### 2.2 Algorithm for rotation matrix between ICS and BCS estimation

Following definition of a rotation matrix, we have$\;C_{I}^{b}=\left[\begin{array}{ccc} {\left[x_{b}\right]}_{I} & {\left[y_{b}\right]}_{I} & {\left[z_{b}\right]}_{I} \end{array}\right]$. The rotation matrix can be estimated by forward rolling the walker along a straight line on a horizontal floor. In this case, the moving direction unit vector d is computed by normalizing the position vector r of the IMU, estimated using the INA (Inertial Navigation Algorithm) as in Section 4, at the end of the straight line. The direction unit vector ${\left[d\right]}_{I}=C_{w}^{I}d$ may not coincide with the x-axis of WCS in ICS ${\left[x_{w}\right]}_{I}$. Let θ be the angle between ${\left[d\right]}_{I}$ and ${\left[x_{w}\right]}_{I}$, so θ can be computed by

(1) $$\{\begin{array}{c} \theta =\cos^{-1}d_{x}\\ \theta =\sin^{-1}d_{y} \end{array}$$

where $d_{x}$, $d_{y}$ and $d_{z}$ are there components of the direction unit vector d.

From the definition of BCS and WCS in Section 2, ${\left[x_{b}\right]}_{I}$ and ${\left[x_{w}\right]}_{I}$ meet at the initial discrete index time. Therefore, ${\left[x_{b}\right]}_{I}$ can be computed by rotating vector ${\left[d\right]}_{I}$ around ${\left[z_{b}\right]}_{I}$ an angle θ, can be found in [25], as follows

(2) $${\left[{\text{x}}_{\text{b}}\right]}_{\text{I}}=\left[\begin{array}{ccc} \cos\text{θ}+{\text{d}}_{\text{x}}^{2}\left(1-\cos\text{θ}\right) & {\text{d}}_{\text{x}}{\text{d}}_{\text{y}}\left(1-\cos\text{θ}\right)-{\text{d}}_{\text{z}}\;\sin\text{θ} & {\text{d}}_{\text{z}}{\text{d}}_{\text{x}}\left(1-\cos\text{θ}\right)+{\text{d}}_{\text{y}}\;\sin\text{θ}\\ {\text{d}}_{\text{y}}{\text{d}}_{\text{x}}\left(1-\cos\text{θ}\right)+{\text{d}}_{\text{z}}\;\sin\text{θ} & \cos\text{θ}+{\text{d}}_{\text{y}}^{2}\left(1-\cos\text{θ}\right) & {\text{d}}_{\text{z}}{\text{d}}_{\text{y}}\left(1-\cos\text{θ}\right)-{\text{d}}_{\text{x}}\;\sin\text{θ}\\ {\text{d}}_{\text{z}}{\text{d}}_{\text{x}}\left(1-\cos\text{θ}\right)-{\text{d}}_{\text{y}}\;\sin\text{θ} & {\text{d}}_{\text{z}}{\text{d}}_{\text{y}}\left(1-\cos\text{θ}\right)+{\text{d}}_{\text{x}}\;\sin\text{θ} & \cos\text{θ}+{\text{d}}_{\text{z}}^{2}\left(1-\cos\text{θ}\right) \end{array}\right]$$

From the definition of BCS in Section 2, we have: the zaxis is pointing upward while the walker is rolling in the horizontal floor. The acceleration measured by the IMU during standing still is the gravitational acceleration. The direction of this acceleration coincides with the z axis of BCS. Thus, z axis of BCS in ICS can be computed by

(3) $${\left[{\text{z}}_{\text{b}}\right]}_{\text{I}}=\frac{{\text{ya}}_{0}}{\left\|{\text{ya}}_{0}\right\|}$$

where ${\text{ya}}_{0}$ is the acceleration measured by the IMU when the walker is standing on the horizontal floor.

Therefore, the rotation matrix ${\text{C}}_{\text{I}}^{\text{b}}$ is computed as follows

(4) $${\text{C}}_{\text{I}}^{\text{b}}=\left[\begin{array}{ccc} {\left[{\text{x}}_{\text{b}}\right]}_{\text{I}} & {\left[{\text{z}}_{\text{b}}\right]}_{\text{I}}\times {\left[{\text{x}}_{\text{b}}\right]}_{\text{I}} & {\left[{\text{z}}_{\text{b}}\right]}_{\text{I}} \end{array}\right]$$

### 2.3 Basic INA using Kalman filter

The basic INA using Kalman filter, can be found in [26, 27], is presented in this section to estimate the walker's trajectory. Let $\text{r}\in {\text{R}}^{3}\;$and $\text{v}\in {\text{R}}^{3}$ are the position and velocity of the IMU in the WCS. Let $\text{C}\left(\text{q}\right)\in {\text{R}}^{3\times 3}$ be the rotation matrix from the WCS to the ICS respectively a quaternion [28] $\text{q}\in {\text{R}}^{4}$.

The quaternion, velocity and position are related as follows

(5) $$\begin{array}{l} \dot{\text{q}}=\frac{1}{2}\left[\begin{array}{ccc} 0 & -{\text{ω}}_{\text{x}} & \begin{array}{cc} -{\text{ω}}_{\text{y}} & -{\text{ω}}_{\text{z}} \end{array}\\ {\text{ω}}_{\text{x}} & 0 & \begin{array}{cc} {\text{ω}}_{\text{z}} & -{\text{ω}}_{\text{y}} \end{array}\\ \begin{array}{c} {\text{ω}}_{\text{y}}\\ {\text{ω}}_{\text{z}} \end{array} & \begin{array}{c} -{\text{ω}}_{\text{z}}\\ {\text{ω}}_{\text{y}} \end{array} & \begin{array}{cc} 0 & {\;\;\;\text{ω}}_{\text{x}}\\ -{\text{ω}}_{\text{x}} & 0 \end{array} \end{array}\right]\text{q}\\ \dot{\text{v}}={\text{C}}^{\text{T}}\left(\text{q}\right){\left[\text{a}\right]}_{\text{I}}\\ \dot{\text{r}}=\text{v} \end{array}$$

where $\text{ω}=\left[\begin{array}{ccc} {\text{ω}}_{\text{x}} & {\text{ω}}_{\text{y}} & {\text{ω}}_{\text{z}} \end{array}\right]$ is the angle rate of the ICS with respect to the WCS and ${\left[\text{a}\right]}_{\text{I}}\in {\text{R}}^{3}$ is the acceleration of IMU with respect to the ICS.

The accelerometer output and gyroscope output (${\text{y}}_{\text{a}}\in {\text{R}}^{3}$ and ${\text{y}}_{\text{g}}\in {\text{R}}^{3}$) are given by

(6) $$\begin{array}{l} {\text{y}}_{\text{a}}={\left[\text{a}\right]}_{\text{I}}+\text{C}\left(\text{q}\right)\tilde{\text{g}}+{\text{v}}_{\text{a}}+{\text{b}}_{\text{a}}\\ {\text{y}}_{\text{g}}=\text{ω}+{\text{v}}_{\text{g}}+{\text{b}}_{\text{g}} \end{array}\text{,}$$

where $v_{a}\in R^{3}$ and $v_{g}\in R^{3}$ are white noise while ${\text{b}}_{\text{a}}\in {\text{R}}^{3}$ and ${\text{b}}_{\text{g}}\in {\text{R}}^{3}$ are biases of accelerometer and gyroscope, respectively. $\tilde{\text{g}}\in {\text{R}}^{3}$ is the local gravitational vector in the WCS.

The numerical integration algorithm to integrate Eq. (5) (replacing ${\left[\text{a}\right]}_{\text{I}}$ with ${\text{y}}_{\text{a}}\;-\;\text{C}\left(\text{q}\right)\tilde{\text{g}}$ and replacing ω with ${\text{y}}_{\text{g}}$) is given in [29]. Let $\hat{\text{q}}$, $\hat{\text{r}}$ and $\hat{\text{v}}$ be the integrated values. There are errors in $\hat{\text{q}}$, $\hat{\text{r}}$ and $\hat{\text{v}}$, due to the sensor noise and they are represented by $\bar{\text{q}}\in {\text{R}}^{3}$, $\bar{\text{r}}\in {\text{R}}^{3}$ and $\bar{\text{v}}\in {\text{R}}^{3}$ as follows

(7) $$\begin{array}{l} \bar{\text{q}}=[\begin{array}{cc} 0_{3\times 1} & {\text{I}}_{3}]\;\left({\hat{\text{q}}}^{\text{*}}⊗\text{q}\right) \end{array}\\ \bar{\text{r}}=\text{r}-\hat{\text{r}}\\ \bar{\text{v}}=\text{v}-\hat{\text{v}} \end{array}\text{,}$$

where ⊗ denotes the quaternion multiplication and ${\text{q}}^{\text{*}}$ is the conjugate quaternion of q.

The state of a Kalman filter is defined by

(8) $$\text{x}=\left[\begin{array}{c} \bar{\text{q}}\\ {\text{b}}_{\text{g}}\\ \begin{array}{c} \bar{\text{r}}\\ \bar{\text{v}}\\ {\text{b}}_{\text{a}} \end{array} \end{array}\right]\in {\text{R}}^{15}$$

The system equation for the Kalman filter is given by:

(9) $$\dot{\text{x}}\left(\text{t}\right)=\text{A}\left(\text{t}\right)\text{x}\left(\text{t}\right)+\text{ω}\left(\text{t}\right)\text{,}$$

Where

$$\text{A}\left(\text{t}\right)=\left[\begin{array}{ccc} \left[-{\text{y}}_{\text{g}}\times \right] & -\frac{1}{2}\text{I} & \begin{array}{cc} 0 & \begin{array}{cc} 0 & 0 \end{array} \end{array}\\ 0 & 0 & \begin{array}{cc} 0 & \begin{array}{cc} 0 & 0 \end{array} \end{array}\\ \begin{array}{c} 0\\ \begin{array}{c} -2{\text{C}}^{\text{T}}\left(\hat{\text{q}}\right)\left[{\text{y}}_{\text{a}}\times \right]\\ 0 \end{array} \end{array} & \begin{array}{c} 0\\ \begin{array}{c} 0\\ 0 \end{array} \end{array} & \begin{array}{cc} 0 & \begin{array}{cc} \text{I} & 0 \end{array}\\ \begin{array}{c} 0\\ 0 \end{array} & \begin{array}{cc} \begin{array}{c} 0\\ 0 \end{array} & \begin{array}{c} 0\\ 0 \end{array} \end{array} \end{array} \end{array}\right],\;\;\text{w}\left(\text{t}\right)=\left[\begin{array}{c} -\frac{1}{2}{\text{v}}_{\text{g}}\\ {\text{w}}_{\text{bg}}\\ \begin{array}{c} 0\\ -{\text{C}}^{\text{T}}\left(\hat{\text{q}}\right){\text{v}}_{\text{a}}\\ {\text{w}}_{\text{ba}} \end{array} \end{array}\right].$$

where $\left[\text{a}\times \right]\in {\text{R}}^{3\times 3}$ is a skew symmetric matrix corresponding to a vector $\text{a}\in {\text{R}}^{3}$. The noises ${\text{w}}_{\text{bg}}$ and ${\text{w}}_{\text{ba}}$ represent small variation of biases.

There are four update equations are analysed in this paper. The first one is the measurement based on zero velocity intervals (ZVIs), and the others are the update equation using the information of encoders.

### 2.4 ZVIs update equation

During walking, there are intervals that the walker is on the horizontal floor and not moving. As can be seen, the velocity and the third component in position (the height) of the walker are almost zero. Therefore, the error in the velocity and the third component of walker can reset.

If the following conditions are satisfied, the discrete time index k is assumed to belong to ZVIs

(10) $$\begin{array}{ll} \left\|{\text{y}}_{\text{g},\text{i}}\right\|\leq {\text{B}}_{\text{g}}, & \text{m}-\frac{{\text{N}}_{\text{g}}}{2}\leq \text{i}\leq \text{m}+\frac{{\text{N}}_{\text{g}}}{2}\\ \left\|{\text{y}}_{\text{a},\text{i}}-{\text{y}}_{\text{a},\text{i}-1}\right\|\leq {\text{B}}_{\text{a}}, & \text{m}-\frac{{\text{N}}_{\text{a}}}{2}\leq \text{i}\leq \text{m}+\frac{{\text{N}}_{\text{a}}}{2} \end{array}$$

where ${\text{N}}_{\text{g}}$ and ${\text{N}}_{\text{a}}$ are integers.

During ZVIs, we have the following zero velocity and position update equations

(11) $$\begin{array}{l} {\text{z}}_{\text{v}}={\text{H}}_{\text{v}}\text{x}+{\text{v}}_{\text{v}}\\ {\text{z}}_{\text{r}}={\text{H}}_{\text{r}}\text{x}+{\text{v}}_{\text{r}} \end{array}$$

where

$$\begin{array}{l} {\text{z}}_{\text{v}}=0_{3\times 1}-\hat{\text{v}}\in {\text{R}}^{3\times 1}\\ {\text{z}}_{\text{r}}=0-\hat{\text{r}}\left(3\right)\\ {\text{H}}_{\text{v}}=\left[\begin{array}{ccc} 0_{3\times 9} & {\text{I}}_{3} & 0_{3\times 3} \end{array}\right]\\ {\text{H}}_{\text{r}}=\left[\begin{array}{ccc} 0_{1\times 8} & {\text{I}}_{1} & 0_{1\times 6} \end{array}\right] \end{array}$$

${\text{v}}_{\text{v}}$ and ${\text{v}}_{\text{r}}$ are white noise reflecting the fact that the velocity and the height of the walker at ZVIs is almost zero.

### 2.5 Quaternion update using vertical vector

The z axis $\left({\text{z}}_{\text{b}}\right)$ of the BCS is upward and coincide with the z axis (${\text{z}}_{\text{w}}$) of the WCS during rolling intervals. This means ${\text{z}}_{\text{w}}$ in the ICS $\left({\left[{\text{z}}_{\text{w},\text{i}}\right]}_{\text{I}}=\text{C}\left({\text{q}}_{\text{i}}\right){\text{z}}_{\text{w}}\right)$ almost does not changed during rolling intervals. Thus, we have

(12) $$\text{C}\left({\text{q}}_{\text{i}}\right){\text{z}}_{\text{w}}={\left[{\text{z}}_{\text{w},1}\right]}_{\text{I}}$$

where ${\text{z}}_{\text{w}}={\left[\begin{array}{ccc} 0 & 0 & 1 \end{array}\right]}^{\text{T}}$ is the z axis of the WCS in the WCS, ${\left[{\text{z}}_{\text{w},1}\right]}_{\text{I}}$ is the z axis of the WCS in the ICS at the discrete time index i=1 (the walker is still not moving).

Due to the feature of quaternion, we have

(13) $$\text{C}\left({\text{q}}_{\text{i}}\right)=\text{C}\left({\hat{\text{q}}}_{\text{i}}\right)-2\text{K}\left({\bar{\text{q}}}_{\text{i}}\right)\text{C}\left({\hat{\text{q}}}_{\text{i}}\right)\text{,}$$

where K(a) of a vector $\text{a}=\left[\begin{array}{ccc} {\text{a}}_{1} & {\text{a}}_{2} & {\text{a}}_{3} \end{array}\right]$ is defined as

$$\text{K}\left(\text{a}\right)=\left[\begin{array}{ccc} 0 & -{\text{a}}_{3} & {\text{a}}_{2}\\ {\text{a}}_{3} & 0 & -{\text{a}}_{1}\\ -{\text{a}}_{2} & {\text{a}}_{1} & 0 \end{array}\right]$$

Insert Eqs. (12) and (13), we have

(14) $$\left[\text{C}\left({\hat{\text{q}}}_{\text{i}}\right)-2\text{K}\left({\bar{\text{q}}}_{\text{i}}\right)\text{C}\left({\hat{\text{q}}}_{\text{i}}\right)\right]{\text{z}}_{\text{w}}={\left[{\text{z}}_{\text{w},1}\right]}_{\text{I}}$$

Rewrite Eq. (14), we have

(15) $$-2\text{K}\left({\bar{\text{q}}}_{\text{i}}\right)\text{C}\left({\hat{\text{q}}}_{\text{i}}\right){\text{z}}_{\text{w}}={\left[{\text{z}}_{\text{w},1}\right]}_{\text{I}}-\text{C}\left({\hat{\text{q}}}_{\text{i}}\right){\text{z}}_{\text{w}}$$

Using K(a)b=−K(b)a (with $\text{a},\text{b}\in {\text{R}}^{3}$) for Eq. (15) we get the measurement equation for quaternion update as follows

(16) $${\text{z}}_{\text{e}1}={\text{H}}_{\text{e}1}\text{x}+{\text{v}}_{\text{e}1}$$

where

$$\begin{array}{c} {\text{z}}_{\text{e}1}={\left[{\text{z}}_{\text{w},1}\right]}_{\text{I}}-\text{C}\left({\hat{\text{q}}}_{\text{i}}\right){\text{z}}_{\text{w}}\\ {\text{H}}_{\text{e}1}=\left[\begin{array}{cc} 2\text{K}\left(\text{C}\left({\hat{\text{q}}}_{\text{i}}\right){\text{z}}_{\text{w}}\right) & 0_{3\times 12} \end{array}\right]\in {\text{R}}^{3\times 15} \end{array},\;$$

the noise ${\text{v}}_{\text{e}1}$ reflecting over the z axis of the BCS which is not really vertical.

### 2.6 Quaternion update using yaw angle

When a walker is moved through wheel rolling, its movement and velocity can be estimated using encoders [30]. Besides, the yaw angle of the walker can be computed and used to derive a measurement update equation for Kalman filter. Let ${\text{E}}_{\text{l},\text{i}}$ and ${\text{E}}_{\text{r},\text{i}}$ be the rolling distance of the left and right wheel in sampling time T at discrete time index i. The distances can be computed by the number of pulses captured from the encoder of the left wheel ${\text{p}}_{\text{l},\text{i}}$ and the right wheel ${\text{p}}_{\text{r},\text{i}}$ as follows

(17) $$\begin{array}{l} {\text{E}}_{\text{l},\text{i}}={\text{λ}}_{\text{l}}{\text{p}}_{\text{l},\text{i}}\\ {\text{E}}_{\text{r},\text{i}}={\text{λ}}_{\text{r}}{\text{p}}_{\text{r},\text{i}} \end{array}$$

where ${\text{λ}}_{\text{l}}$ and ${\text{λ}}_{\text{r}}$ are factors related with the resolution of encoder (the number of pulses per round) and the radius of the left and right wheel.

**Fig. 3:**
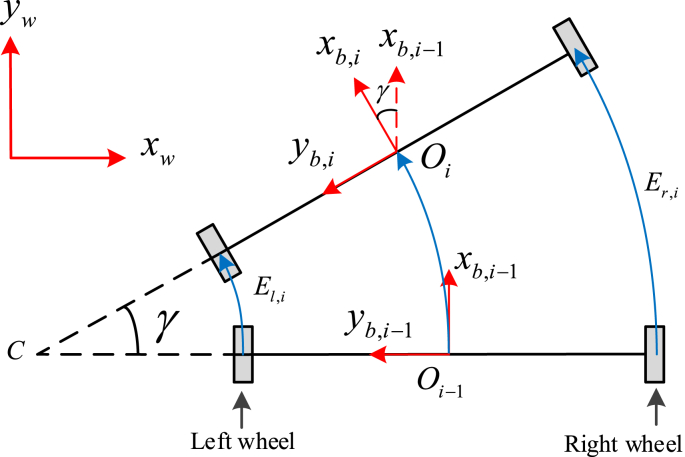
Position and angle computation during rolling intervals.

Since the distance between two wheels (D=610mm) is very much larger than the rolling distances of wheels ( ${\text{E}}_{\text{l},\text{i}},{\;\;\text{E}}_{\text{r},\text{i}}\approx 5\;\text{mm}$) in each sampling time T, the movement of walker can be considered as a rotation movement around a center C during discrete time index i in rolling case (see Fig. 3). The yaw angle at discrete time index i can be computed as follows

(18) $${\text{γ}}_{\text{i}}=\frac{{\text{E}}_{\text{l},\text{i}}-{\text{E}}_{\text{r},\text{i}}}{\text{D}}$$

The rotation matrix from BCS at discrete time index i to BCS at i−1 is

(19) $${\text{C}}_{\text{b},\text{i}-1\;}^{\text{b},\text{i}}=\left[\begin{array}{ccc} \cos{\text{γ}}_{\text{i}} & -\sin{\text{γ}}_{\text{i}} & 0\\ \sin{\text{γ}}_{\text{i}} & \cos{\text{γ}}_{\text{i}} & 0\\ 0 & 0 & 1 \end{array}\right]$$

Thus, the translation vector from BCS at discrete time index i to BCS at i−1 can be computed

(20) $${\left[{\text{T}}_{\text{b},\text{i}-1}^{\text{b},\text{i}}\right]}_{\text{b},\text{i}-1}={\text{C}}_{\text{b},\text{i}-1\;}^{\text{b},\text{i}}{\left[\begin{array}{ccc} \frac{{\text{E}}_{\text{l},\text{i}}+{\text{E}}_{\text{r},\text{i}}}{2} & 0 & 0 \end{array}\right]}^{T}$$

The translation vector in WCS is

(21) $${\text{T}}_{\text{b},\text{i}-1}^{\text{b},\text{i}}={\text{C}}_{\text{b},\text{i}-1}^{\text{w}}{\text{C}}_{\text{b},\text{i}-1\;}^{\text{b},\text{i}}{\left[\begin{array}{ccc} \frac{{\text{E}}_{\text{l},\text{i}}+{\text{E}}_{\text{r},\text{i}}}{2} & 0 & 0 \end{array}\right]}^{T}$$

Insert ${\text{C}}_{\text{b},\text{i}-1}^{\text{w}}={\text{C}}^{\text{T}}\left({\hat{\text{q}}}_{\text{i}-1}\right){\text{C}}_{\text{b}}^{\text{I}}$ to Eq. (21), we have

(22) $${\text{T}}_{\text{b},\text{i}-1}^{\text{b},\text{i}}={\text{C}}^{\text{T}}\left({\hat{\text{q}}}_{\text{i}-1}\right){\text{C}}_{\text{b}}^{\text{I}}{\text{C}}_{\text{b},\text{i}-1\;}^{\text{b},\text{i}}{\left[\begin{array}{ccc} \frac{{\text{E}}_{\text{l},\text{i}}+{\text{E}}_{\text{r},\text{i}}}{2} & 0 & 0 \end{array}\right]}^{T}$$

Therefore, the position of walker at discrete time index i can be computed by the position at i−1 with respect to WCS as follows

(23) $${\text{r}}_{\text{b},\text{i}}={\text{r}}_{\text{b},\text{i}-1}+{\;\text{T}}_{\text{b},\text{i}-1}^{\text{b},\text{i}}$$

Furthermore, the position of walker and IMU are related by

(24) $$\begin{array}{c} {\text{r}}_{\text{b},\text{i}}={\text{r}}_{\text{i}}-{\text{C}}_{\text{b},\text{i}}^{\text{w}}{\left[{\text{T}}_{\text{b}}^{\text{I}}\right]}_{\text{b}}\\ {\text{r}}_{\text{i}}={\text{r}}_{\text{b},\text{i}}+{\text{C}}_{\text{b},\text{i}}^{\text{w}}{\left[{\text{T}}_{\text{b}}^{\text{I}}\right]}_{\text{b}} \end{array}$$

As can be seen in Fig. 3 the x axis of BCS at i (${\text{x}}_{\text{b},\text{i}}$) is the rotation of the x axis of BCS at i−1 (${\text{x}}_{\text{b},\text{i}-1}$) around the z axis of BCS an angle ${\text{γ}}_{\text{i}}$, we have

(25) $${\left[{\text{C}}_{\text{I}}^{\text{b}}\text{C}\left({\text{q}}_{\text{i}}\right)\right]}^{\text{T}}{\left[\begin{array}{ccc} 1 & 0 & 0 \end{array}\right]}^{\text{T}}={\text{C}}_{\text{b},\text{i}-1\;}^{\text{b},\text{i}}\left({\left[{\text{C}}_{\text{I}}^{\text{b}}\text{C}\left({\hat{\text{q}}}_{\text{i}-1}\right)\right]}^{\text{T}}{\left[\begin{array}{ccc} 1 & 0 & 0 \end{array}\right]}^{\text{T}}\right)$$

where ${\text{C}}_{\text{I}}^{\text{b}}={\left[{\text{C}}_{\text{b}}^{\text{I}}\right]}^{\text{T}}$.

Let $\text{RE}={\text{C}}_{\text{b},\text{i}-1\;}^{\text{b},\text{i}}\left({\left[{\text{C}}_{\text{I}}^{\text{b}}\text{C}\left({\hat{\text{q}}}_{\text{i}-1}\right)\right]}^{\text{T}}{\left[\begin{array}{ccc} 1 & 0 & 0 \end{array}\right]}^{\text{T}}\right)$ and use $\text{C}\left({\text{q}}_{\text{i}}\right)=\text{C}\left({\hat{\text{q}}}_{\text{i}}\right)-2\text{K}\left({\bar{\text{q}}}_{\text{i}}\right)\text{C}\left({\hat{\text{q}}}_{\text{i}}\right)$, we have

(26) $${\left[{\text{C}}_{\text{I}}^{\text{b}}\left(\text{C}\left({\hat{\text{q}}}_{\text{i}}\right)-2\text{K}\left({\bar{\text{q}}}_{\text{i}}\right)\text{C}\left({\hat{\text{q}}}_{\text{i}}\right)\right)\right]}^{\text{T}}{\left[\begin{array}{ccc} 1 & 0 & 0 \end{array}\right]}^{\text{T}}=\text{RE}$$

Rewrite Eq. (26), we have

(27) $${\left[{\text{C}}_{\text{I}}^{\text{b}}\text{C}\left({\hat{\text{q}}}_{\text{i}}\right)-2{\text{C}}_{\text{I}}^{\text{b}}\text{K}\left({\bar{\text{q}}}_{\text{i}}\right)\text{C}\left({\hat{\text{q}}}_{\text{i}}\right)\right]}^{\text{T}}{\left[\begin{array}{ccc} 1 & 0 & 0 \end{array}\right]}^{\text{T}}=\text{RE}$$

Apply ${\left(\text{ab}\right)}^{\text{T}}={\text{b}}^{\text{T}}{\text{a}}^{\text{T}}$ to Eq. (27), we have

(28) $$\left({\left[{\text{C}}_{\text{I}}^{\text{b}}\text{C}\left({\hat{\text{q}}}_{\text{i}}\right)\right]}^{\text{T}}-2{\left[\text{K}\left({\bar{\text{q}}}_{\text{i}}\right)\text{C}\left({\hat{\text{q}}}_{\text{i}}\right)\right]}^{\text{T}}{\left[{\text{C}}_{\text{I}}^{\text{b}}\right]}^{\text{T}}\right){\left[\begin{array}{ccc} 1 & 0 & 0 \end{array}\right]}^{\text{T}}=\text{RE}$$

Apply ${\left(\text{ab}\right)}^{\text{T}}={\text{b}}^{\text{T}}{\text{a}}^{\text{T}}$ and ${\left[\text{K}\left(\text{a}\right)\right]}^{\text{T}}=-\text{K}\left(\text{a}\right)$ to Equaiton (28), we have

(29) $${\left[{\text{C}}_{\text{I}}^{\text{b}}\text{C}\left({\hat{\text{q}}}_{\text{i}}\right)\right]}^{\text{T}}{\left[\begin{array}{ccc} 1 & 0 & 0 \end{array}\right]}^{\text{T}}+2{\text{C}}^{\text{T}}\left({\hat{\text{q}}}_{\text{i}}\right)\text{K}\left({\bar{\text{q}}}_{\text{i}}\right){\left[{\text{C}}_{\text{I}}^{\text{b}}\right]}^{\text{T}}{\left[\begin{array}{ccc} 1 & 0 & 0 \end{array}\right]}^{\text{T}}=\text{RE}$$

Using ${\left[\text{K}\left(\text{a}\right)\right]}^{\text{T}}=-\text{K}\left(\text{a}\right)$ for Equaiton (29), we have

(30) $$-2{\text{C}}^{\text{T}}\left({\hat{\text{q}}}_{\text{i}}\right)\text{K}\left({\left[{\text{C}}_{\text{I}}^{\text{b}}\right]}^{\text{T}}{\left[\begin{array}{ccc} 1 & 0 & 0 \end{array}\right]}^{\text{T}}\right){\bar{\text{q}}}_{\text{i}}=\text{RE}-{\left[{\text{C}}_{\text{I}}^{\text{b}}\text{C}\left({\hat{\text{q}}}_{\text{i}}\right)\right]}^{\text{T}}{\left[\begin{array}{ccc} 1 & 0 & 0 \end{array}\right]}^{\text{T}}$$

Thus, the measurement update equation for quaternion using yaw angle during rolling is

(31) $${\text{z}}_{\text{e}2}={\text{H}}_{\text{e}2}\text{x}+{\text{v}}_{\text{e}2}$$

where

$${\text{z}}_{\text{e}2}=\text{RE}-{\left[{\text{C}}_{\text{I}}^{\text{b}}\text{C}\left({\hat{\text{q}}}_{\text{i}}\right)\right]}^{\text{T}}{\left[\begin{array}{ccc} 1 & 0 & 0 \end{array}\right]}^{\text{T}},$$

$${\;\text{H}}_{\text{e}2}=\left[\begin{array}{cc} -2{\text{C}}^{\text{T}}\left({\hat{\text{q}}}_{\text{i}}\right)\text{K}\left({\left[{\text{C}}_{\text{I}}^{\text{b}}\right]}^{\text{T}}{\left[\begin{array}{ccc} 1 & 0 & 0 \end{array}\right]}^{\text{T}}\right) & 0_{3\times 12} \end{array}\right]\in {\text{R}}^{3\times 15},$$

the noise ${\text{v}}_{\text{e}2}$ reflect the error in yaw angle computation.

### 2.7 Position update using encoders

During rolling intervals of the walker, we can use information of encoders to compute the position of the walker. The computed position can be used to form measurement update equation for Kalman filter.

From Eq. (24) we have

(32) $${\text{r}}_{\text{i}}={\text{r}}_{\text{b},\text{i}}+{\text{C}}_{\text{b},\text{i}-1}^{\text{w}}{\left[{\text{C}}_{\text{b},\text{i}-1}^{\text{b},\text{i}}\right]}^{\text{T}}{\left[{\text{T}}_{\text{b}}^{\text{I}}\right]}_{\text{b}}$$

Since ${\text{C}}_{\text{b},\text{i}-1}^{\text{w}}={\text{C}}^{\text{T}}\left({\hat{\text{q}}}_{\text{i}-1}\right){\text{C}}_{\text{b}}^{\text{I}}$, we have

(33) $${\text{r}}_{\text{i}}={\text{r}}_{\text{b},\text{i}}+{\text{C}}^{\text{T}}\left({\hat{\text{q}}}_{\text{i}-1}\right){\text{C}}_{\text{b}}^{\text{I}}{\left[{\text{C}}_{\text{b},\text{i}-1}^{\text{b},\text{i}}\right]}^{\text{T}}{\left[{\text{T}}_{\text{b}}^{\text{I}}\right]}_{\text{b}}$$

Insert ${\text{r}}_{\text{b},\text{i}}$ in Eqs. (23), (24), (25), (26), (27), (28), (29), (30), (31), (32) and (33), we have

(34) $${\text{r}}_{\text{i}}={\text{r}}_{\text{b},\text{i}-1}+{\;\text{T}}_{\text{b},\text{i}-1}^{\text{b},\text{i}}+{\text{C}}^{\text{T}}\left({\hat{\text{q}}}_{\text{i}-1}\right){\text{C}}_{\text{b}}^{\text{I}}{\left[{\text{C}}_{\text{b},\text{i}-1}^{\text{b},\text{i}}\right]}^{\text{T}}{\left[{\text{T}}_{\text{b}}^{\text{I}}\right]}_{\text{b}}$$

Apply ${\text{r}}_{\text{b},\text{i}}={\text{r}}_{\text{i}}-{\text{C}}_{\text{b},\text{i}}^{\text{w}}{\left[{\text{T}}_{\text{b}}^{\text{I}}\right]}_{\text{b}}$ to discrete time index i−1, we have

(35) $${\text{r}}_{\text{b},\text{i}-1}={\text{r}}_{\text{i}-1}-{\text{C}}_{\text{b},\text{i}-1}^{\text{w}}{\left[{\text{T}}_{\text{b}}^{\text{I}}\right]}_{\text{b}}$$

Replace ${\text{C}}_{\text{b},\text{i}-1}^{\text{w}}$ by ${\text{C}}^{\text{T}}\left({\hat{\text{q}}}_{\text{i}-1}\right){\text{C}}_{\text{b}}^{\text{I}}\;$for Eq. (35), we have

(36) $${\text{r}}_{\text{b},\text{i}-1}={\text{r}}_{\text{i}-1}-{\text{C}}^{\text{T}}\left({\hat{\text{q}}}_{\text{i}-1}\right){\text{C}}_{\text{b}}^{\text{I}}{\left[{\text{T}}_{\text{b}}^{\text{I}}\right]}_{\text{b}}$$

Thus, Eq. (34) can be rewritten as follows

(37) $${\text{r}}_{\text{i}}={\text{r}}_{\text{i}-1}-{\text{C}}^{\text{T}}\left({\hat{\text{q}}}_{\text{i}-1}\right){\text{C}}_{\text{b}}^{\text{I}}{\left[{\text{T}}_{\text{b}}^{\text{I}}\right]}_{\text{b}}+{\;\text{T}}_{\text{b},\text{i}-1}^{\text{b},\text{i}}+{\text{C}}^{\text{T}}\left({\hat{\text{q}}}_{\text{i}-1}\right){\text{C}}_{\text{b}}^{\text{I}}{\left[{\text{C}}_{\text{b},\text{i}-1}^{\text{b},\text{i}}\right]}^{\text{T}}{\left[{\text{T}}_{\text{b}}^{\text{I}}\right]}_{\text{b}}$$

In which components in right hand side are already computed at discrete time index i−1.

(38) $${\text{z}}_{\text{e}3}={\text{H}}_{\text{e}3}\text{x}+{\text{v}}_{\text{e}3}$$

where

$$\begin{array}{c} {\text{z}}_{\text{e}3}={\text{r}}_{\text{i}}-{\hat{\text{r}}}_{\text{i}}\\ {\text{H}}_{\text{e}3}=\left[\begin{array}{ccc} 0_{3\times 9} & {\text{I}}_{3} & 0_{3\times 3} \end{array}\right]\in {\text{R}}^{3\times 15} \end{array}$$

The noise ${\text{v}}_{\text{e}3}$ refects the error of position computation.

## 3 Experimental

**Fig. 4:**
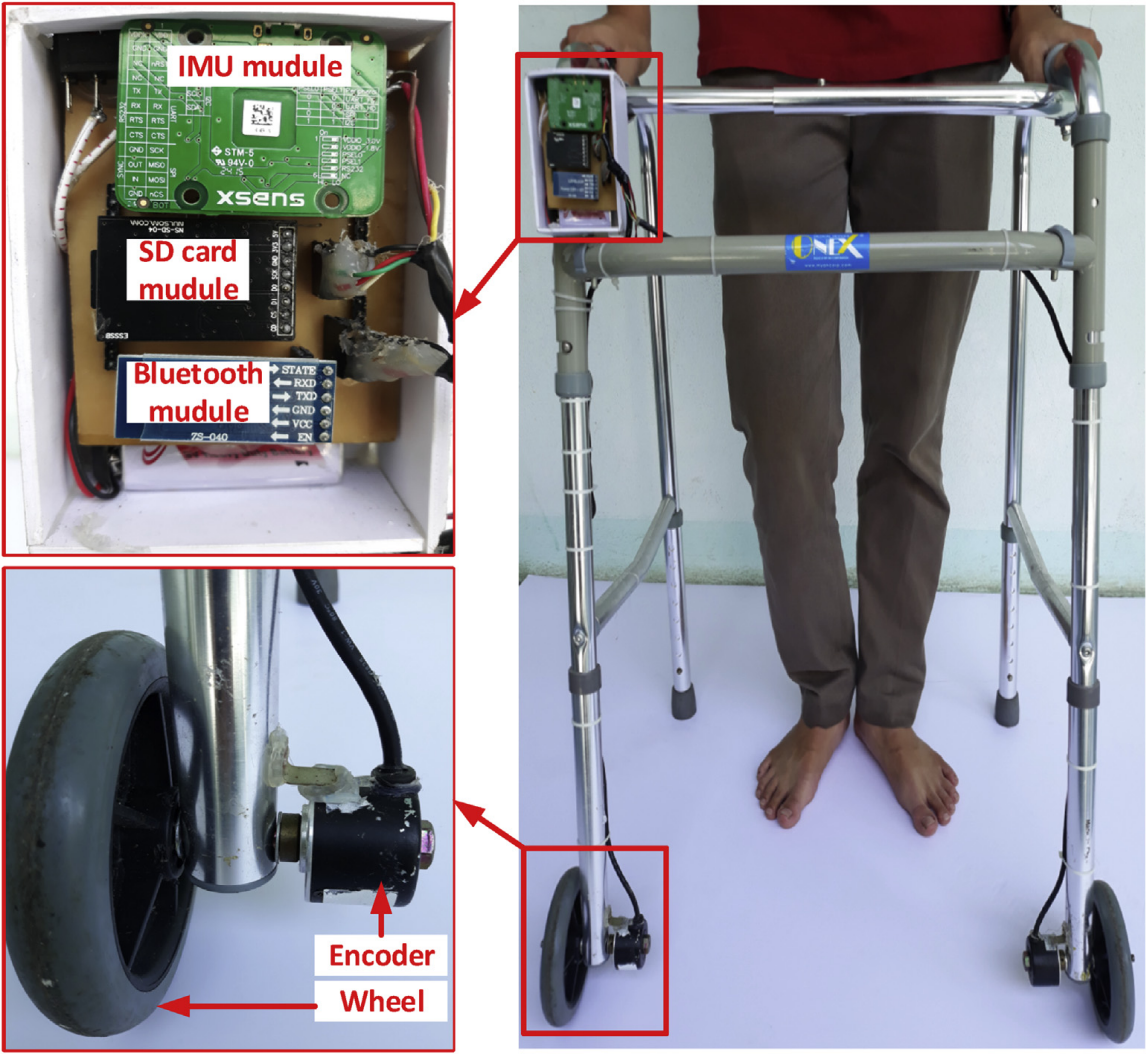
Proposed system for walking monitoring.

The walker system for experiments to evaluate the accuracy of the proposed algorithm is shown in Fig. 4. In which, the frequency of an IMU (Xsens Mti-1) is 100 Hz and the resolution of encoders is 1024 ppr. The walking parameters estimation, movement detection and classification algorithm for user have been mentioned in Section V and VI [16]. In this paper, we focus on the accuracy and the effect of three proposed update equation in Section 5.

Five volunteers taking part in the experiment are asked to walk 20 m along the corridor using the walker. Each person walks 20 times consisting of 5 times for continuous rolling, 5 times for step by step rolling, 5 times for complete lifting and 5 times for 2-back tips lifting (walking style definition, detection and classification can be found in [16]). The volunteers are introduced how to use the walker as the older. The results of the experiment are shown in Table 1 (for continuous rolling), Table 2 (for step by step rolling), Table 3 (for complete lifting) and Table 4 (for 2-back tips lifting).

**Table 1:** Distance estimation error (m) for 20 m walking with continuous rolling.

User	Standard	Pure INA	INA + vertical update	INA + yaw angle update	INA + position update
1	Mean	−4.594	−7.664	0.597	0.312
	RMSE	5.002	7.724	5.359	0.313
2	Mean	−42.362	−4.390	−9.892	0.328
	RMSE	51.243	4.514	14.518	0.340
3	Mean	−9.278	−4.540	0.425	0.156
	RMSE	35.987	4.648	17.964	0.210
4	Mean	2.702	6.928	−2.175	0.200
	RMSE	2.835	7.006	17.857	0.237
5	Mean	6.848	−3.946	11.255	0.640
	RMSE	7.417	3.999	12.152	0.730
***Sum***	***Mean***	***−9.337***	***−5.493***	***0.242***	***0.327***
	***RMSE***	***28.316***	***5.774***	***14.344***	***0.412***

Mean: Mean of error. RMSE: Root mean square of error.

**Table 2:** Distance estimation error (m) for 20 m walking with step-by-step rolling.

User	Standard	Pure INA	INA + vertical update	INA + yaw angle update	INA + position update
1	Mean	−0.438	0.537	0.069	0.1880
	RMSE	0.453	0.558	0.078	0.194
2	Mean	−0.443	0.010	0.045	−0.23
	RMSE	0.444	0.113	0.099	0.239
3	Mean	−0.719	−0.321	−0.316	−0.272
	RMSE	0.738	0.365	0.346	0.282
4	Mean	−0.303	0.242	0.023	0.024
	RMSE	0.309	0.260	0.076	0.084
5	Mean	−0.319	0.170	0.023	−0.002
	RMSE	0.332	0.207	0.087	0.137
***Sum***	***Mean***	***−0.445***	***0.128***	***−0.030***	***−0.059***
	***RMSE***	***0.480***	***0.337***	***0.173***	***0.200***

Mean: Mean of error. RMSE: Root mean square of error.

**Table 3:** Distance estimation error (m) for 20 m walking with complete lifting.

User	Standard	Pure INA	INA + vertical update	INA + yaw angle update	INA + position update
1	Mean	0.231	0.369	0.473	0.467
	RMSE	0.275	0.397	0.495	0.499
2	Mean	0.510	0.629	0.510	0.520
	RMSE	0.573	0.677	0.562	0.559
3	Mean	−0.292	−0.158	−0.062	0.005
	RMSE	0.544	0.477	0.448	0.438
4	Mean	0.248	0.334	0.401	0.452
	RMSE	0.397	0.449	0.497	0.536
5	Mean	−0.460	−0.350	−0.262	−0.194
	RMSE	0.504	0.405	0.332	0.282
***Sum***	***Mean***	***0.047***	***0.164***	***0.212***	***0.250***
	***RMSE***	***0.471***	***0.492***	***0.473***	***0.473***

Mean: Mean of error. RMSE: Root mean square of error.

**Table 4:** Distance estimation error (m) for 20 m walking with 2 back tips lifting.

User	Standard	Pure INA	INA + vertical update	INA + yaw angle update	INA + position update
1	Mean	0.360	0.481	0.566	0.383
	RMSE	0.399	0.513	0.595	0.441
2	Mean	0.712	0.623	0.903	0.299
	RMSE	0.729	0.699	0.915	0.441
3	Mean	0.060	0.121	0.174	0.218
	RMSE	0.076	0.132	0.183	0.225
4	Mean	0.237	0.317	0.379	0.325
	RMSE	0.311	0.383	0.440	0.381
5	Mean	0.060	0.149	0.219	0.272
	RMSE	0.152	0.205	0.262	0.309
***Sum***	***Mean***	***0.286***	***0.338***	***0.448***	***0.299***
	***RMSE***	***0.404***	***0.437***	***0.545***	***0.369***

Mean: Mean of error. RMSE: Root mean square of error.

In each walking style, we show the mean of error and the root mean square of error (RMSE) of 20 m walking distance estimation using the pure INA (see column 3), quaternion update using vertical vector (see column 4), quaternion update using yaw angle (see column 5) and position update using encoders (see column 6). Meanwhile the mean value is used to show the center of the distance error and the RMSE shows the difference between distance errors. In this case, the RMSE value is more important than mean value.

As can be seen in Tables 1 and 2, the proposed update equations significant improve the accuracy of distance estimation in continuous rolling case (the RMSE reduces from 28.316 m to 0.412 m and the mean of error reduces from −9.337 m to 0.327 m). However, there is a slight improvement in the accuracy using the proposed update equations in the step by step rolling case (the RMSE reduces from 0.480 m to 0.200 m and the mean of error reduces from −0.445 m to −0.059 m). In this situation, the INA using the position update equation gives the best results. In the lifting case (Tables 3 and 4), there is no improvement using update equation because the information of encoders is not used to update the estimation of trajectory in lifting case.

Besides, comparing the results in column 4 and 5 of Tables 1 and 2 we reveals that the INA using the vertical vector update gives better results than the INA using the yaw angle update in the long movement. But the INA using the yaw angle update gives good results in the short movement (note that the movement in step by step rolling case can be separated by short movements).

## 4 Conclusions

In this paper, we propose a system for trajectory of walker estimation. The system consists of an inertial sensor and two encoders attached to a front-wheel walker. The inertial sensor is used to estimate the trajectory of the walker while the encoders are used to update the trajectory of the walker during rolling on the floor.

In this paper, three proposed update equations are proposed: quaternion update using the vertical vector, quaternion update using the yaw angle of the walker and position update using encoders. We implemented an experiment focusing on four walking styles using the walker: continuous rolling case, step by step rolling case, complete lifting case and 2 back lips lifting case. Results of the experiment show the appropriateness of proposed update equations in all cases in general and in continuous rolling in particular.

The proposed update equations significantly improve the accuracy of distance estimation in continuous rolling case (the RMSE reduces from 28.316 m to 0.412 m and the mean of error reduces from −9.337 m to 0.327 m). However, there is a slightly improvement in the accuracy using the proposed update equations in the step by step rolling case (the RMSE reduces from 0.480 m to 0.200 m and the mean of error reduces from −0.445 m to −0.059 m). In this paper, the INA using the position update equation gives the best results. In the lifting case, there is no improvement using update equation because the information of encoders is not use to update for the trajectory estimation in lifting case.

In our future work, a module Bluetooth is integrated in the walker system to transfer the recorded data to a mobile phone. Furthermore, a user's app for a mobile is built to transfer the data to a host machine using the Internet and get the walking parameter and doctor's advices from health care centre. By this way, a user can get data at home without doctor using the walker system by himself and send the data to a health care centre. User walking parameters will be computed using the proposed algorithm in host machine at the centre. With the help of these parameters, a doctor can evaluate the user's health and give some useful advice for user via user's account.

## Declarations

### Author contribution statement

Quang Vinh Doan: Analyzed and interpreted the data; Contributed reagents, materials, analysis tools or data.

Duy Duong Pham: Conceived and designed the experiments; Performed the experiments; Wrote the paper.

### Funding statement

This work is supported by the Ministry of Education and Training (MOET), Vietnam under project No. B2018.DNA.07 (KYTH-45).

### Competing interest statement

The authors declare no conflict of interest.

### Additional information

No additional information is available for this paper.
